# pH-Responsive Elastin-Like Polypeptide Designer Condensates

**DOI:** 10.1021/acsami.3c11314

**Published:** 2023-09-14

**Authors:** Robbert J. de Haas, Ketan A. Ganar, Siddharth Deshpande, Renko de Vries

**Affiliations:** Department of Physical Chemistry and Soft Matter, Wageningen University and Research, 6708 WE Wageningen, The Netherlands

**Keywords:** liquid−liquid phase separation, biomolecular condensates, elastin-like polypeptides, pH-responsive coacervation, synthetic cells

## Abstract

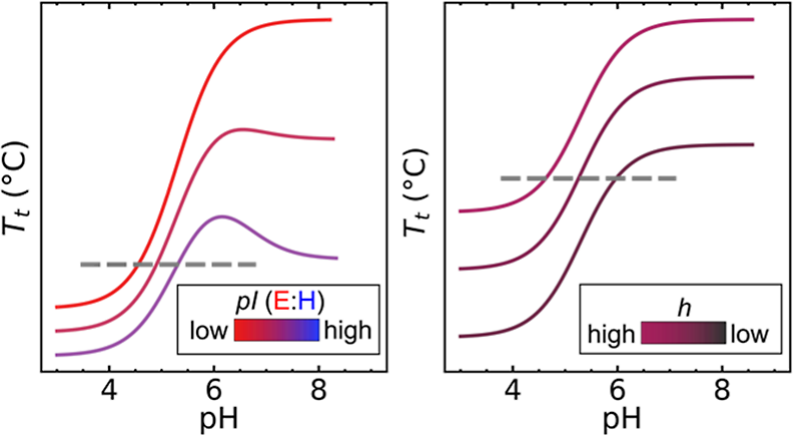

Biomolecular condensates are macromolecular complexes formed by liquid–liquid phase separation. They regulate key biological functions by reversibly compartmentalizing molecules in cells, in a stimulus-dependent manner. Designing stimuli-responsive synthetic condensates is crucial for engineering compartmentalized synthetic cells that are able to mimic spatiotemporal control over the biochemical reactions. Here, we design and test a family of condensate-forming, pH-responsive elastin-like polypeptides (ELPs) that form condensates above critical pH values ranging between 4 and 7, for temperatures between 20 and at 37 °C. We show that the condensation occurs rapidly, in sharp pH intervals (ΔpH < 0.3). For eventual applications in engineering synthetic cell compartments, we demonstrate that multiple types of pH-responsive ELPs can form mixed condensates inside micron-sized vesicles. When genetically fused with enzymes, receptors, and signaling molecules, these pH-responsive ELPs could be potentially used as pH-switchable functional condensates for spatially controlling biochemistry in engineered synthetic cells.

## Introduction

Biomolecular condensates are dense assemblies of biomolecules that form via liquid–liquid phase separation (LLPS). In cells, condensates are abundantly present and regulate crucial biological functions such as growth, metabolism, and reproduction via compartmentalization of key biomolecules and spatiotemporally organizing biochemical reactions.^1^ For example, compartmentalization of DNA and translation-associated proteins is central to rRNA synthesis and ribosome biogenesis in prokaryotes.^2,3^ Crucial to cell survival, such condensates often respond to environmental changes in temperature, pH, or solute concentrations. For example in yeast, a pH decrease leads to the formation stress-granular condensates that act as a molecular ON switch for the translation of stress-survival proteins.^4^

However, cells are too complex to decipher these processes completely in vivo. A useful and complementary approach is to study these interactions using model systems, preferably within cell-mimicking confinements, without interference from other cellular systems.^5^ This is also a first step toward the in vitro synthetic biology of creating nature-inspired synthetic assemblies for biomedical and biotechnological applications.^6–8^ Technological advances have made such controlled bioengineering studies more possible than ever, complementing the in vivo studies.^5,7,9^

Condensates of low-complexity polypeptide domains such as elastin-like polypeptides (ELPs)^10^ and resilin-like polypeptide (RLP)^11^ have emerged as important model systems to understand and design protein-based condensates through simple coacervation. They are already playing an increasingly important role in engineering synthetic cells, where control over compartmentalization is a key challenge.^12–14^

In current ELP-based systems, the temperature is used as the main control parameter for inducing and reversing condensation. The ELPs feature (GXGVP) pentapeptide repeats, where the condensate transition temperature depends strongly on the identity of the typically hydrophobic guest residue X. For engineering stimulus-responsive compartmentalization in synthetic cells, as well as for other applications, it would be very useful to have genetically encoded polypeptides that form condensates responding not only to temperature but also to chemical stimuli, such as pH. Indeed, synthetic polymers with tunable pH-responsive condensation have been designed and these are being used in applications such as drug delivery to slightly acidic tumors or pH quantitation during endocytosis.^15,16^

While it is well-known that for ELPs with charged residues, condensation can be induced by pH changes,^17–19^ a systematic study of tuning ELP condensation via pH by varying ELP sequence has not yet been performed. With this in mind, we proposed to design a family of pH-responsive, condensate forming ELPs (PREs), with critical pH values in the physiologically relevant range of 4–7.

We did so by precisely tuning the fractions of charged and hydrophobic guest residues in the ELPs and found that condensation occurred rapidly above the critical pH values, in sharp pH intervals (ΔpH < 0.3). As a demonstration of the possible usefulness of these polymers in engineering functional compartments in confined spaces, we demonstrated the formation of mixed, pH-sensitive condensates inside micron-sized synthetic compartments. Ultimately, pH-sensitive compartmentalization within synthetic cells could be used to carry out multiple spatially separated biochemical reactions simultaneously. The work we present here is a first step in that direction.

## Results and Discussion

**Figure 1:**
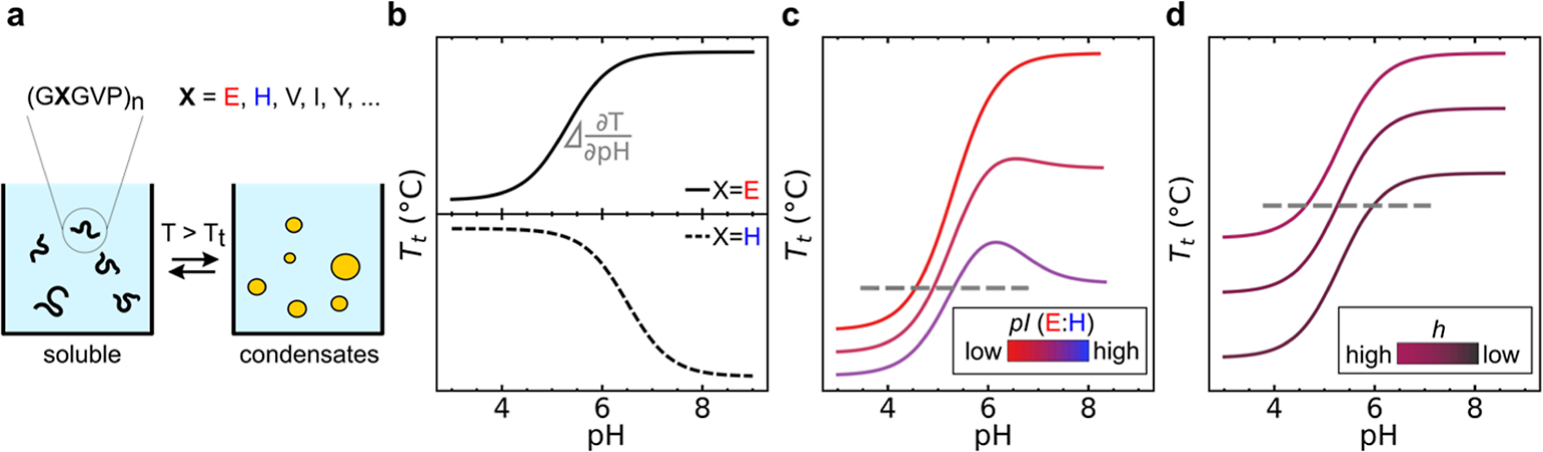
(a) ELPs are water-soluble protein polymers (black) and can form condensates (yellow) if the temperature increases beyond its LCST, referred to as the transition temperature (*T*_t_). ELPs have a general sequence (GXGVP)_*n*_, where X can be any amino acid (except proline) and *n* is the length of the polymer. Upon reaching the *T*_t_, ELPs desolvate and form condensate droplets that eventually coalesce into larger condensates. Typically condensation occurs within a temperature change of <3 °C. (b) Schematic plots based on MacKay et al. model of the phase behavior of recombinant pH-responsive ELPs (eq 1).^17^ pH-responsive ELPs (PREs) can be made by introducing guest residues with side groups that can protonate such as negatively charged glutamic acid (E; Glu) or positively charged histidine (H; His). The ∂*T*/∂pH is the steepest near the isoelectric point (pI) of the PRE that is determined by the identity of the charged guest residue composition. (c) Schematic plots of *T*_t_ versus pH for PREs where guest residue contains a combination of E and H at various ratios (with H/E always below 1), and thus various pI. Under the right conditions, these PREs can be engineered to condense at different pHs at constant temperature (see gray dashed line). (d) Schematic plot of *T*_t_ versus pH for PREs with a fixed number of E and H, but varying in hydrophobicity, *h* (Urry hydrophobicity index). At a fixed temperature, PREs can be engineered to condense at different pH values (gray dashed line).

ELPs display lower critical solution temperature (LCST) phase separation behavior, wherein upon a temperature increase beyond a critical threshold, the ELPs rapidly desolvate and phase separate and form condensates^11,20^ (Figure 1a). This transition temperature (*T*_t_) can be precisely tuned by concentration, salt, ELP length, and guest residue identity.^11,20^

pH-responsive ELPs (PREs) can be designed by including charged guest residues, such as glutamic acid (Glu; E) or histidine (His; H). Charge improves the solvation of the polymer, making it less favorable to collapse and form condensates.^18^ PREs with guest residues Glu (p*K*_a_ 4.1) or His (p*K*_a_ 6.0) have shown to exhibit a *T*_t_ that depends strongly on protonation state, and thus pH.^17^ The pH-dependence of *T*_t_ follows a Henderson–Hasselbach like profile, where naively one expects the inflection pH (pH_i_) to be close to the polymer isoelectric point, or pI (Figure 1b).^17^

The pH-response change ∂*T*/∂pH near the pH_i_ dictates the smallest temperature difference required to trigger condensation by pH. For PREs with guest residue Glu this was found to be approximately 23 °C per pH unit.^17^ Since temperature-triggered ELP condensation typically occurs over a temperature windows of a few degrees, Δ*T* ∼ 3 °C,^20,21^ we estimated that a pH-trigged PRE condensation (at constant temperature) can switch within ∼0.13 pH units. This should allow for the design of PREs that respond to very small pH changes or gradients.

The pH sensitivity of PREs can be tuned by length and concentration, but a systematic study of tuning condensation via pH by varying the protein sequence has not been performed. We hypothesized that the PRE condensation at different pH values can be precisely engineered through sequence design.

To achieve this, one strategy is to engineer PREs with proper amounts of Glu and His guest residues. For example, by increasing the relative ratio of histidine to glutamic acids, the PRE pI can be shifted to higher values. We expected that under the right conditions this would lead to a shift in the *T*_t_ versus pH plots, such that at a constant temperature, PREs with distinctly different pH transitions (pH_t_) at constant temperature could be designed (Figure 1c).

A second strategy is to tune the PRE hydrophobicity by including a larger fraction of pentamers with hydrophobic guest residues.^20^ We expected that by tuning the hydrophobicity, a regime can be found at constant temperature that gives rise to PREs that form condensates at different pH_t_ (Figure 1d). As a measure for PRE hydrophobicity *h*, we will use Urry’s hydrophobicity scale that was specifically designed to rank-order guest residue hydrophobicity in ELPs.^18^ A low *h* means a PRE will form a condensate at lower temperature and vice versa.

### PREs with Varying Glu and His Residue Ratios

We first explored the strategy of engineering PREs with guest residues varying in the E and H ratios. We chose E/H ratios [4:0], [3:2] and [2:3] ensuring equal spacing of charged guest residues along the polymer sequence. Similar to previous model ELPs, the PREs also contain a certain fraction of pentamers with valine (V) and isoleucine (I) as guest residues, in order to increase the hydrophobicity and bring *T*_t_ values down to the relevant range. We denote this first series as PRE-pI-*x*, where *x* denotes the theoretical pI of the (isolated) guest residues predicted from the primary sequence using Expasy.^22^ PRE-pI-*x* sequences are summarized in Table 1 and are shown in full in Table S1. The PRE length was kept constant at 60 pentamers, such that the molar weights were equal at ∼27 kDa.

**Table 1:** PRE-pI-*x* Series of Polymers with Varying Amounts of Glutamic Acid (E) and Histidine (H) Residues^a^

design name	protein sequence
PRE-pI-3.2	(GXGVP)_60_, with X = V/I/E/H [4:12:4:0]
PRE-pI-4.7	(GXGVP)_60_, with X = V/I/E/H [5:10:3:2]
PRE-pI-5.7	(GXGVP)_60_, with X = V/I/E/H [5:10:2:3]

a Relative amounts of the 4 types of guest residues X are given in square brackets.

For the PRE-pI-*x* series, we obtained synthetic genes encoding 20 pentapeptides and constructed plasmids with 60 pentapeptides by two successive rounds of PRE-RDL cloning.^23^ The PREs were recombinantly expressed in *Escherichia coli* and purified by two rounds of inverse thermal cycling (ITC).^24^ The final purified proteins showed no signs of degradation (Figure S1). PREs were dialyzed in milli-Q, lyophilized, and stored at −20 °C until use.

We studied the condensation behavior of PRE-pI-*x* polymers by measuring the turbidity while gradually increasing the temperature to a protein concentration of 25 μM. We did so in buffered solutions for a large number of pH values. For pH > 6, we used 50 mM phosphate + 100 mM NaCl buffers, abbreviated as PBS^100^. For pH < 6, we used 50 mM succinate + 100 mM NaCl, abbreviated as SBS^100^. These two buffers were chosen in view of their low temperature sensitivity.^17^

**Figure 2:**
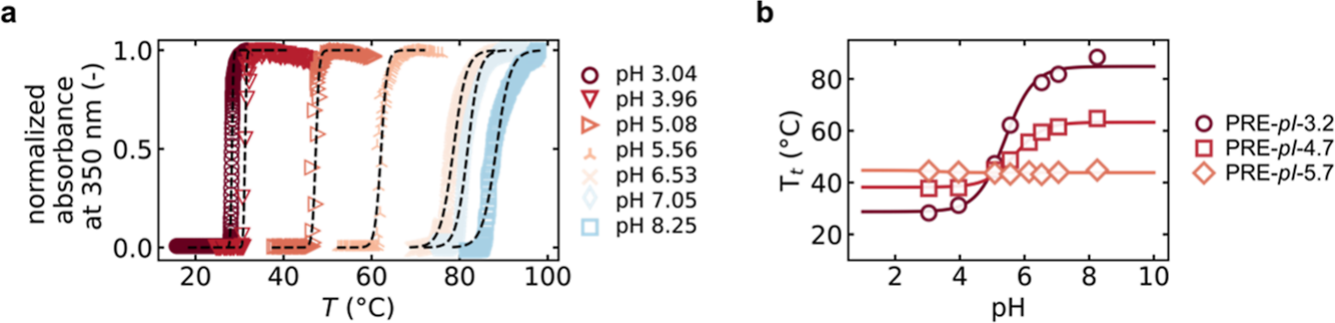
Characterization of the pH dependence of PRE phase transitions. Polymer concentration 25 μM, buffer PBS^100^ (pH > 6) or SBS^100^ (pH < 6). (a) Turbidity of PRE-pI-3.2 at constant pH plotted against a temperature at 1 °C/min. Temperature-trigged condensation is rapid and usually takes ∼3 °C. Black dotted lines are sigmoidal fits used to determine temperature transition *T*_t_ at the inflection point. (b) *T*_t_ vs pH for the PRE-pI-*x* polymers with various values of *x*. The pH inflection point changes minimally with the increased pI (due to the higher ratio of His/Glu). A higher number of His residues decreased the *T*_t_ range, with the case of PRE-pI-5.7 demonstrating the limit. Just tuning Glu/His ratio in PREs is insufficient to realize PREs with strong and sharp pH-induced transitions.

To illustrate the method, Figure 2a shows representative turbidity measurements for PRE-pI-3.2 at various pH values. Upon condensation, the turbidity increased rapidly. We defined the transition temperature (*T*_t_) as the inflection point of a sigmoidal fit to the turbidity data. Figure 2b shows an overview of the pH dependence of *T*_t_ obtained for the entire PRE-pI-*x* series.

The observed pH_i_ for PRE-pI-3.2, which comprises solely Glu as charged residues, was 5.3, exhibiting a substantial deviation from the predicted pI of 3.2. This discrepancy has been observed by other authors,^17,18^ and we speculate that the local chemical environment of Glu in the polymer changes the degree of (de)protonation of its side chain. Similarly, for PRE-pI-4.7 we found an increased pH_i_ of 5.7.

Interestingly, we find that increasing the *x* value further reduces the *T*_t_ range and the sharpness ∂*T*/∂pH near the inflection point. As a limiting case, for PRE-pI-5.7, condensation is found to be completely pH-insensitive, despite this PRE having many His and Glu residues (Figure 2b). Possibly, PREs with a ratio of E/H [2:3] could be used to design condensates with varying net charge at different pH values, for example, for the pH-dependent accumulation of charged guest molecules. Clearly, tuning only the Glu/His ratio is insufficient to realize PREs with strong and sharp pH-induced transitions.

### PREs with Varying Hydrophobicity

Next, we explored tuning the pH_t_ of PREs by changing the overall hydrophobicity of PREs (Figure 1d). We designed a series of 6 polymers, PRE-*h*-*x* with different average Urry hydrophobicity *x*, but at a fixed Glu to His ratio (E/H [4:1]). Hydrophobicity was tuned by changing the guest residue valine to isoleucine (V/I) ratio, as well as by introducing specific guest residues with a high Urry hydrophobicity, such as phenylalanine (F) and tyrosine (Y), to substitute for isoleucine (I) (Tables 2 and S1).

**Table 2:** PRE-*h*-*x* Series Polymers with a Fixed Fraction of E and H Guest Residues of [4:1] and Varying Fractions of Hydrophobic Guest Residues^a^

design name	protein sequence
PRE-*h*-58	(GXGVP)_60_, with X = V/I/E/H [2:13:4:1]
PRE-*h*-46	(GXGVP)_60_, with X = V/F/E/H [7:8:4:1]
PRE-*h*-43	(GXGVP)_60_, with X = V/F/E/H [6:9:4:1]
PRE-*h*-41	(GXGVP)_60_, with X = V/F/E/H [5:10:4:1]
PRE-*h*-36	(GXGVP)_60_, with X = V/Y/E/H [7:8:4:1]
PRE-*h*-35	(GXGVP)_60_, with X = V/F/E/H [3:12:4:1]

a *x* is the overall Urry hydrophobicity, *h* of the polymers. Relative amounts of the 4 types of guest residues X are given in square brackets.

Similar to the PRE-pI-*x* series, synthetic genes were obtained and concatemerized using PRE-RDL cloning and recombinantly purified using ITC purification (Figure S1). PRE-*h*-*x* was dialyzed in milli-Q, lyophilized, and stored at −20 °C until use.

**Figure 3:**
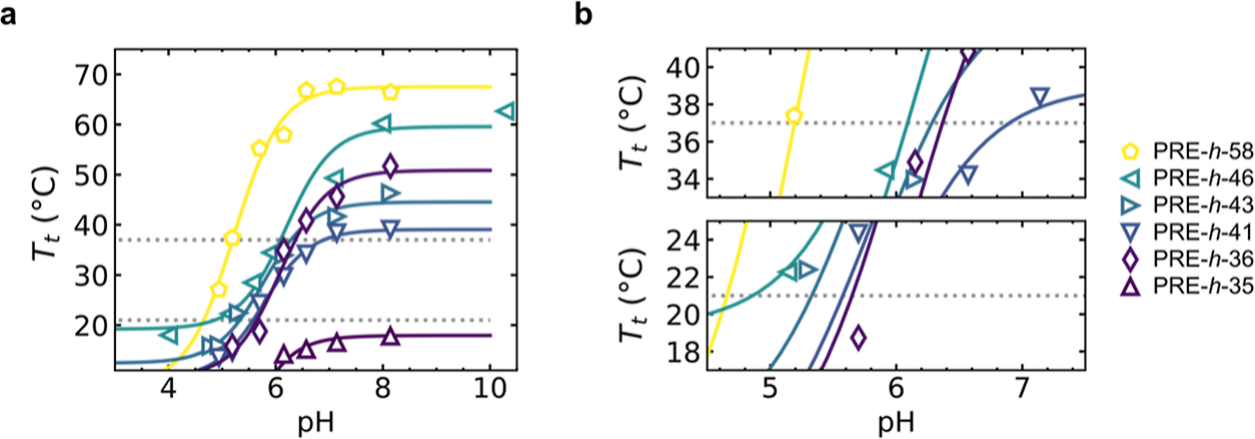
(a) *T*_t_ vs pH profiles for PRE-*h*-*x* polymers. Solid lines are sigmoidal fits. Transition temperatures of the PRE-*h*-*x* designs qualitatively follow the Urry hydrophobicity (*h*), with the exception of PRE-*h*-36 that contains multiple tyrosines in the guest residue composition (Table 2). Dotted gray lines are 37 °C and room temperature (21 °C). (b) Zoom for temperatures 37 °C (top) and 21 °C (bottom).

Figure 3a shows the *T*_t_ vs pH plots for PRE-*h*-*x* series at 25 μM. As expected, the increasing hydrophobicity decreased the solubility, and qualitatively follows the *h* index. The only exception to this trend is PRE-*h*-36 that contained several tyrosines (Y) at the guest residue positions. We speculate that similar to what we observed for PRE-pI-3.2, the local environment of the tyrosine alters its protonation (p*K*_a_ 10.5), such that its hydroxyl group can be protonated at lower pH, which would drastically increase its solubility.^18^

**Figure 4:**
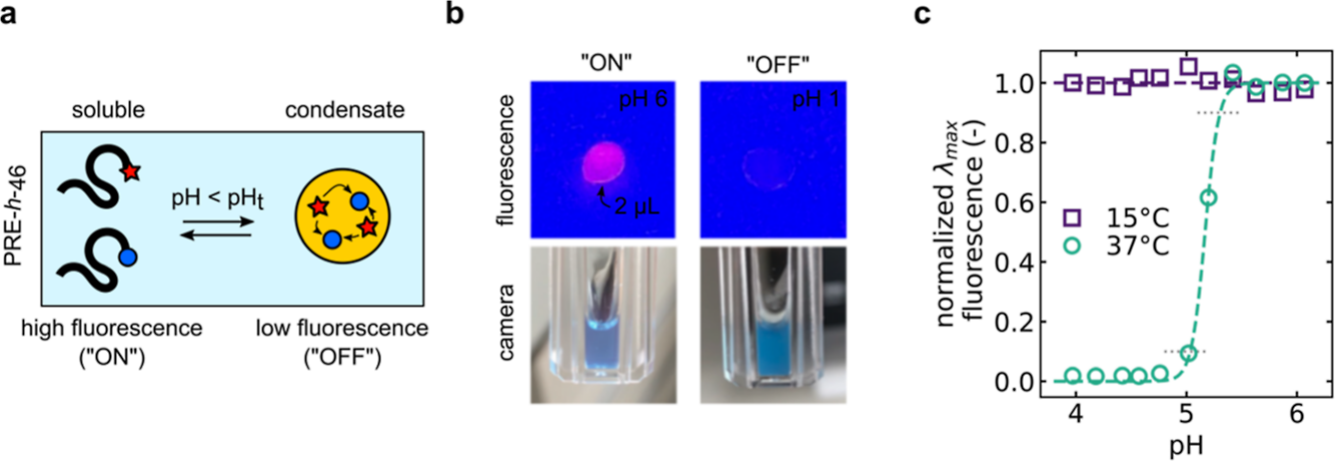
Sharpness of pH transitions demonstrated by using PRE-*h*-46 as a fluorescent pH ON/OFF switch. (a) Schematic overview of PRE-*h*-46 labeled near the c-terminal with AT532 dye (red) and AT612Q quencher (blue), above and below the condensation threshold (pH_t_). Condensation brings the dye and quencher in close proximity leading to fluorescence quenching and a decrease in the fluorescence signal (“OFF” state). (b) Top: images of a 2 μL droplet of 25 μM AT532-PRE-*h*-46 + AT612Q-PRE-*h*-46 in SBS^100^ buffer on a UV transilluminator plate at pH 6 (“ON”) and pH 1 (“OFF”). Bottom: camera images of cuvettes: at pH 1, the fluorescence completely disappears, and the solution turns turbid as a result of PRE condensation. (c) Normalized AT590 fluorescence intensity at λ_max_ = 650 nm plotted over pH for the 25 μM AT532-PRE-*h*-46 + AT612Q-PRE-*h*-46 mixture in PBS^140^. Each data point was dialyzed to the indicated pH (0.2 pH unit steps). Fluorescence peaks were obtained at 15 and 37 °C (after 5 min equilibration). At 15 °C, the PRE-*h*-46 remains soluble under all pH conditions, and the fluorescence does not switch (Figure 3b). At 37 °C, the condensation occurs at pH_t_ = 5.2 with a sharpness of approximately 0.3 pH units between the “ON” and “OFF” state.

As relevant temperatures for which we wanted to obtain polymers with varying pH_t_ values, we chose the range between 21 and 37 °C. From Figure 4b we observe that with the PRE-*h*-*x* series we can achieve pH_t_ in the range of pH 4 to pH 7 for this temperature range. This means that at isothermal conditions, the PRE-*h*-*x* can switch at programmed pH values. The pH_t_ values for each of the PRE-*h*-*x* at 21 and 37 °C are summarized in Table 3.

**Table 3:** Transition pH Values (pH_t_) at Room Temperature (21 °C) and 37 °C, for 25 μM PRE-*h*-*x* Polymers in SBS^100^ (for pH < 6) or PBS^100^ (for pH > 6)

design name	pH_t_^21 °C^	pH_t_^37 °C^
PRE-*h*-58	4.6	5.2
PRE-*h*-36	5.7	6.4
PRE-*h*-46	5.1	6.1
PRE-*h*-43	5.4	6.3
PRE-*h*-41	5.6	6.9
PRE-*h*-35	N/A	N/A

### Sharpness of pH Transitions

Having established a library of PREs with a defined pH_t_, we set out to validate our hypothesis that pH_t_ condensation should occur over a narrow pH range. To demonstrate this, using the PRE-*h*-46 polymer, we designed a fluorescent pH-switch. We did so by bioconjugating an ATTO-590 (AT590) fluorescent dye and an ATTO-612Q (AT612Q) quencher to separate batches of the PRE-*h*-46 polymers. In a mixture of AT590-PRE-*h*-46 and AT612Q-PRE-*h*-46, the formation of a condensate brings the dye and quencher close such that quenching through resonance energy transfer takes place, which reduces fluorescence signal from high state (“ON”) to low state (“OFF”) (Figure 4a).

A mixture of the AT590-PRE-*h*-46 and AT612Q-PRE-*h*-46 polymers (25 μM each) was dialyzed (at 4 °C) into SBS^140^ buffers of various pH values. As a first test, we used HCl to decrease the pH of the SBS^140^ pH 6 buffer to pH ∼ 1 and measured fluorescence of a 2 μL droplet under a UV illuminator. We observed a bright pink, fluorescent signal for the sample still at pH 6, and a dark signal, barely showing the contours of the droplet, for the acidified sample at pH ∼ 1. Simultaneously, we visually observed that the acidified solution indeed turned cloudy due to PRE condensation (Figure 4b). Next, we recorded fluorescent spectra in a range of pH 4–6, with increments of 0.2 pH units at 15 °C (far below the expected pH_t_ of PRE-*h*-46, for any pH, see Figure 3a) and at 37 °C degrees. Upon increasing the temperature from 15 to 37 °C, condensation occurred, bringing together the dye and quencher that led to efficient quenching of >50 fold (Figure S2). Figure 4C shows the fluorescence emission peak against the pH. We observe that the “ON/OFF” switch indeed occurs over a narrow pH range, approximately ΔpH < 0.3 for a change from 90 to 10% of fluorescent intensity.

Based on Table 3, for PRE-*h*-46, we would have expected a pH_t_ = 6.0 at 37 °C. Instead, we found pH_t_ = 5.2, about 0.8 pH units lower. Although the ionic strength of the buffer SBS^140^ is comparable to SBS^100^ used previously, a higher [NaCl] in SBS^140^ increased the overall *T*_t_ likely due to higher [Na^+^] ions.^25^ This is supported by Figure S3, which shows that an increase of 10 °C in *T*_t_ was measured for PRE-*h*-43 in SBS^140^ compared to SBS^100^. This relatively strong dependence on [Na^+^] is likely to make implementation of pH sensors for in vivo applications using the PREs challenging. On the other hand, we confirmed that the influence of PRE concentration on the *T*_t_ is relatively weak (Figure S4), suggesting experimental differences in protein concentration quantitation are likely not the cause of this discrepancy.

### Engineering Mixed pH-Sensitive Condensates in Micron-Sized Compartments

**Figure 5:**
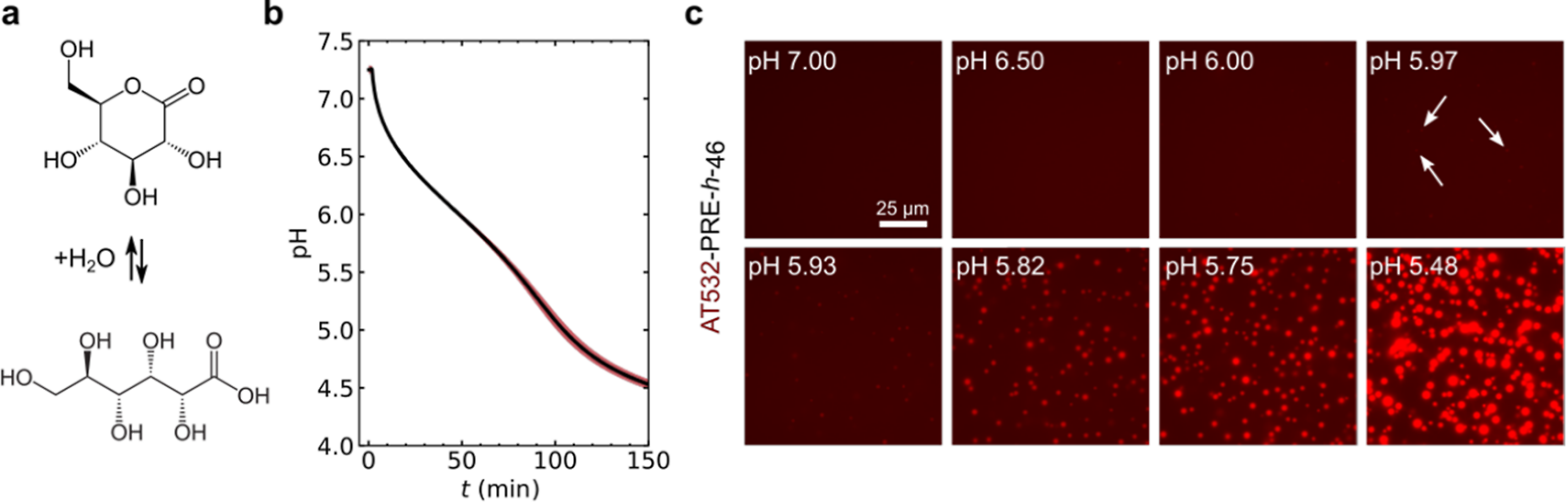
(a) GDL hydrolyzes into gluconic acid, which leads to slow and steady acidification over time. (b) pH calibration was performed upon addition of 15 mg/mL GDL to PBS^100^. The solid black line shows average of three independent calibrations. Red area shows the standard deviation. (c) Time-lapse fluorescence images of AT532-labeled PRE-*h*-46 at different pH values. At pH 5.97, the first condensates can be observed (a few are indicated by white arrows). Condensates continue to coalesce and grow. Scale bar: 25 μm.

Having validated that the PREs have sharp pH intervals, we sought to study the phase behavior at microscopic resolution in real-time and at isothermal temperature. To do so, we used glucono-δ-lactone (GDL), which hydrolyses into gluconic acid over time, to create well-defined pH-time profiles (Figure 5a,b). First, we performed a bulk calibration experiment of GDL in PBS^100^ at room temperature. Figure 3b shows an average pH calibration curve for 3 independent measurements of GDL hydrolysis over time, with a maximal pH standard deviation of 0.07 pH units.

Next, at room temperature, we followed the condensation of a 25 μM solution of PRE-*h*-46 doped with 5% fluorescent AT590-PRE-*h*-46 by fluorescence microscopy. Figure 5c shows selected fluorescence microscopy images of the pH-induced condensation process. We found condensation occurs over a very narrow pH window of ΔpH < 0.06 (between pH 5.97 and pH 5.93). Hence, microscopically, the condensation transition was even sharper than that inferred through turbidity measurements.

Through microscopic visualization, we found a pH_t_ ∼ 5.8 at 21 °C, where in bulk according to Table 3, a pH_t_ of ∼5.1 was expected. This 0.7 pH unit mismatch between bulk and microscopic sample can be explained by the fact that, in the bulk, turbidity started to become measurable only when condensate droplets coalesced to large enough size. For pH-triggered condensation the coalescence was relatively slow (Figure S5), while condensates of ∼1 μm in size were readily detected through microscopy.

Next, we considered what happens during acidification for paired PREs with similar and different pH_t_ values. To visualize the condensate droplets, we chose to fluorescently label PRE-*h*-41, PRE-*h*-36, and PRE-*h*-58 with FITC, Cy5, and Cy3 respectively. Previously it was found that ELPs of the same length with guest residues X = A/V that differ >20% in guest residue content formed multilayer condensates.^26^ We, therefore, paired (i) PRE-*h*-41 + PRE-*h*-36 and (ii) PRE-*h*-36 + PRE-*h*-58 at 25 μM each (doped with 10% labeled variants) and studied their condensation behavior during GDL acidification by microscopy at room temperature. Note both pairs were significantly different at the sequence level: the PRE-*h*-41 + PRE-*h*-36 pair was 50% different and the PRE-*h*-36 + PRE-*h*-58 pair was even 75% different in guest residue composition (see Table 2).

For these two pairs, Figure 3b shows their expected pH_t_ values at room temperature. PRE-*h*-36 and PRE-*h*-41 are highly similar at pH_t_ ∼ 5.5 and, thus, predicted to condense at the same pH, while the pair PRE-*h*-41 + PRE-*h*-36 has different pH_t_ and condensation is expected to be sequential.

**Figure 6:**
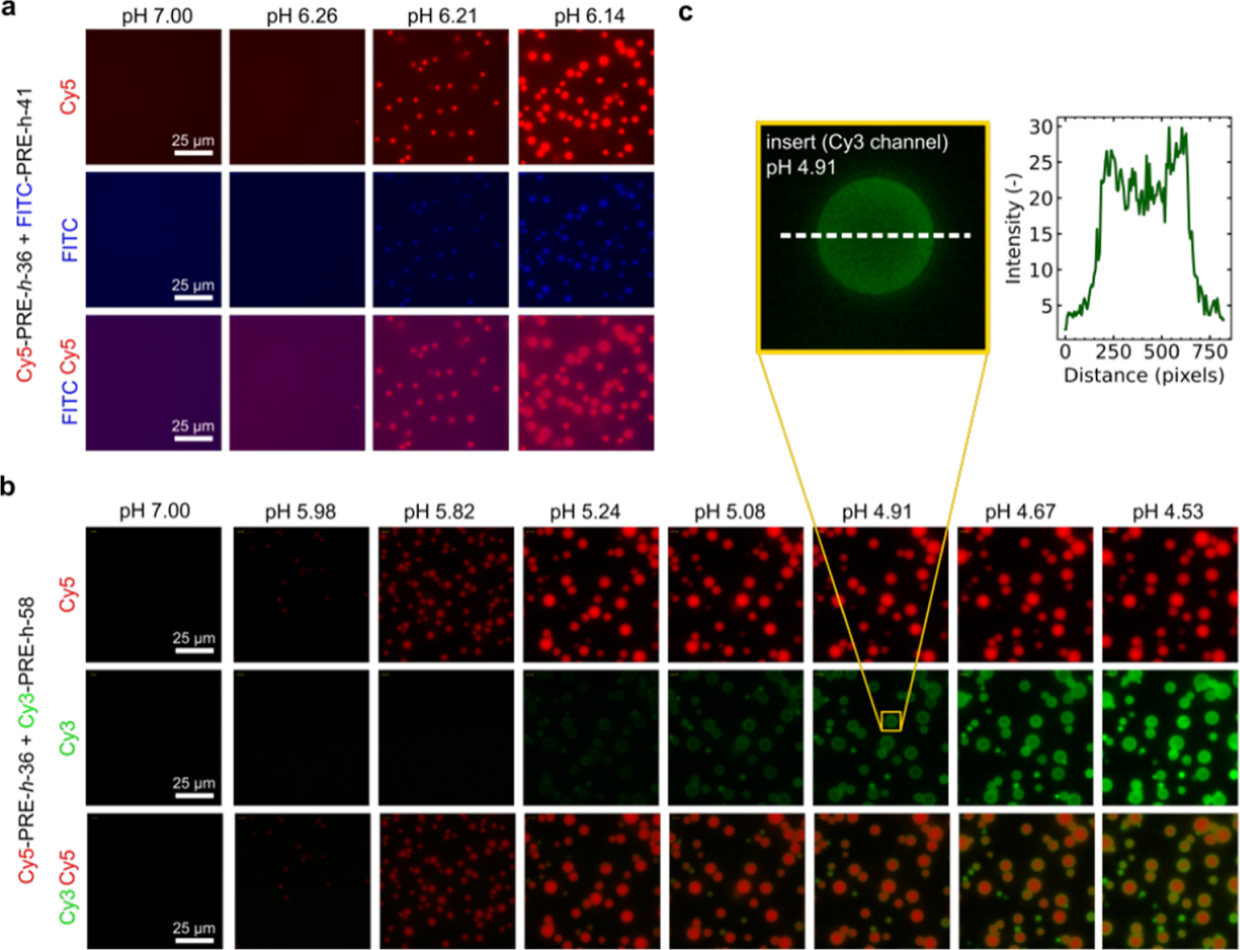
Sequential (mixed) condensate formation at the microscale at room temperature. (a) Mixtures of 25 μM Cy5-PRE-*h*-36 and 25 μM FITC-PRE-*h*-41 in PBS^100^ acidified with 15 mg/mL of GDL at different time/pH points. From previous turbidity experiments, PRE-*h*-41 and PRE-*h*-36 were expected to form condensates at the same pH (see Figure 3b). The pair formed mixed condensates starting at pH 6.21. No clear signs of multiphase condensation were observed in FITC and Cy5 fluorescent channels. Scale bar: 25 μm. (b) Mixtures of 25 μM Cy5-PRE-*h*-36 and Cy3-PRE-*h*-58 in PBS^100^ acidified with 15 mg/mL GDL at different time/pH points. From previous turbidity experiments Cy5-PRE-*h*-36 and Cy3-PRE-*h*-58 were expected to form condensates at the same pH (Figure 3b) and this was indeed the case: Cy5-PRE-*h*-36 first formed condensates at pH 5.82, followed by Cy3-PRE-*h*-58 at pH 5.24. Interestingly Cy3-PRE-*h*-58 appeared to wet the surface of Cy5-PRE-*h*-36 condensates and ultimately formed mixed condensates. Scale bar: 25 μm. (c) Insert of wetting behavior of Cy3-PRE-*h*-58 at pH 4.91 in the Cy3 channel. The pixel intensity of the white dashed line was quantified and plotted, showing a clear accumulation of intensity at the edges of the condensate. “Cy5, FITC, Cy5” indicates the fluorescent channel at which data were recorded. Combinations “FITC Cy5” and “Cy3 Cy5” indicate overlaid/merged data.

Figure 6a shows condensation for PRE-*h*-36 and PRE-*h*-41 pairs indeed occurred at the same time and shows condensation starting at pH_t_ ∼ 6.2. The condensates coalesce and mix because of the PRE sequences of PRE-*h*-36 and PRE-*h*-41, contrary to the expectation.

Figure 6b shows the condensation of the PRE-*h*-36 and PRE-*h*-58 pair. Here, it was expected that PRE-*h*-36 condensed first and thereafter PRE-*h*-58. This is indeed observed in the experiments. PRE-*h*-36 condensation started at pH 6.14, while for PRE-*h*-58, condensation started at pH 5.24. Interestingly, we found that PRE-*h*-58 condensates wetted the surfaces of the preformed PRE-*h*-36 condensates, before eventually forming the mixed condensate droplets (Figure 6c).

While PRE-*h*-36 and PRE-*h*-58 display substantial differences in their guest residue composition (Table 2), they do not undergo demixed condensate formation, unlike ELPs with less variation in the guest residue composition that have shown such behavior.^26^ For the PREs to form mixed condensates, there must be some form of attraction between the two PREs, such as pi–pi stacking or electrostatic interactions.^27^ To create multiphase PRE condensates, we anticipate that further altering the sequence compositions and the molecular weight of the PREs will be necessary.^27^

**Figure 7:**
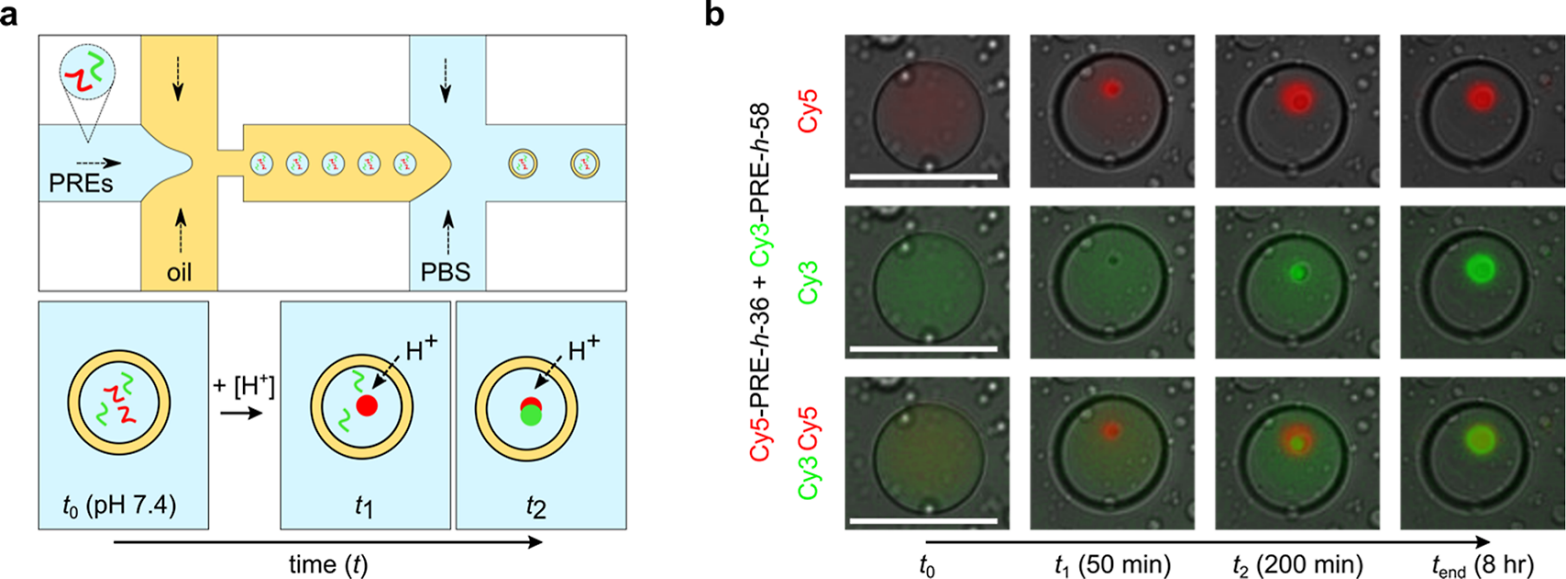
Sequential (mixed) condensate formation in microcompartments at room temperature. (a) Top: schematic of microfluidic production of double-emulsion droplets with different types of fluorescently labeled PRE polymers. Bottom: acidification is induced by the addition of PBS^100^ (pH 2) in the external environment. Acidification occurs slowly over time due to proton flux across the oil membrane. Depending on their pH_t_, different ELP species will phase separate at different times (*t*_1_ and *t*_2_), possibly leading to mixed condensates in the end. (b) Fluorescence stills of droplets containing Cy5-PRE-*h*-36 and Cy3-PRE-*h*-58 at 25 μM each acidified over time with time points *t*_0_, *t*_1_, *t*_2_, and *t*_end_ indicated. Cy5 and Cy3 as well as Cy5 and Cy5 overlapped/merged fluorescent channels are shown. At *t*_end_, a fully mixed condensate is observed. Scale bar: 50 μm.

Lastly, to show the potential of such pH-sensitive ELP condensates for compartmentalizing synthetic cells, we demonstrated sequential condensation in cell-sized confinements. Double emulsion (water–oil–water) droplets were produced using microfluidics, encapsulating a mixture of 25 μM Cy5-PRE-*h*-36 and Cy3-PRE-*h*-58 in PBS^100^ inside (Figures 7a and S6). Next, we added PBS^100^ at pH 2 in the external environment to eventually decrease the pH inside the emulsions through the proton flux across the double emulsion boundary. Because of the thin oil boundary of the vesicles, proton transport occurs gradually, similar to GDL hydrolysis over time. As shown in Figure 7a, we expected to first observe condensation of Cy5-PRE-*h*-36 inside the microcompartments at some time *t*_1_, and this should be followed by condensation of Cy3-PRE-*h*-58 at some later time *t*_2_. Indeed, as shown in Figure 7b, we found Cy5-PRE-*h*-36 condensed first and Cy3-PRE-*h*-58 condensed later within the microcompartments. Ultimately, as was also observed in bulk experiments, both condensates mix to form one single hybrid condensate. Some small droplets can be observed in the background. These are unwanted oil droplets formed during the early stages when the production is not yet optimized, or satellite droplets formed along with double emulsions, or a result of bursting of some of the double emulsions during the experiment. These oil droplets remain outside of the double-emulsions and do not interfere with our proof-of-concept experiment (Figure 7b). Full field-of-view of many such double-emulsion droplets are shown in Figure S7.

## Conclusions

In conclusion, by engineering pH-responsive ELPs that we coin PREs, we have designed a library of condensates that can be switched “ON/OFF” by small pH changes in the range of pH 4–7. This range is significant, as it covers the pH range found in many biological systems, including the cytoplasm and endosomes. Our PRE design is based on systematically including charged and increasingly hydrophobic guest residues in the ELP sequence and, therefore, expands on the previous work of López et al. concerning the design of ELPs with mixed or multiphase condensates in cell-like confinements.^26^ Moreover, our approach takes a significant further step by incorporating pH-controlled switches for condensation in addition to temperature sensitivity.

The design of these PREs opens up a range of possibilities for simulating biological compartmentalization. Such a protein sequence design approach, combined with microfluidic technology, can prove useful to understand the way natural cells form multiple, coexisting membraneless organelles in a dynamic fashion and to produce complex synthetic cells in the future. Given the recent surge of interest in forming coacervate-based organelles in biomimetic vesicles,^28–33^ the presented pH-responsive ELP condensates will likely prove handy to the synthetic cell community. Ultimately, the pH-responsive nature of these condensates can be utilized to spatially and temporally regulate the function of enzymes, receptors, and signaling molecules, allowing for a highly precise control over biological processes in synthetic cells.

## Experimental Section

### Plasmid Construction

Codon optimized synthetic gene fragments (Twist Bioscience), encoding 20 ELP pentapeptide repeats (100 amino acids), were cloned into a modified pET-24(+) vector using *Bam*HI and XhoI restriction endonucleases (New England Biolabs). Two successive rounds of recursive directional ligation by plasmid reconstruction (PRe-RDL) were performed to construct genes of 60 ELP repeats (300 aa). Expression plasmids encoding ELPs were Sanger sequenced to ensure the correct protein sequence and transformed into T7-Express *E. coli* (New England Biolabs) via heat-shock.

### Recombinant Protein Purification

A single colony was used to inoculate a 25 mL Terrific Broth starter culture, supplemented with 50 mg L^–1^ kanamycin (Sigma-Aldrich). The starter culture is grown overnight at 37 °C in a shaker and used to inoculate 1 L of LB containing 10 g of tryptone (Bacto), 10 g of NaCl (Sigma-Aldrich), and 5 g of yeast extract (Bacto), supplemented with 50 mg L^–1^ kanamycin. The culture was incubated shaking until 0.6 < OD_600_ < 0.8 at 37 °C in a 2 L baffled Erlenmeyer. Protein expression was induced by 1 mM isopropyl β-d-thiogalactoside (IPTG) and expression was continued at 18 °C overnight. The cell broth was centrifuged and 6000*g* and the cell pellet was resuspended in 4 °C 30 mL of PBS^140^ pH 7.4 (10 mM phosphate, 140 mM NaCl) with 1 mM phenylmethylsulfonyl fluoride (PMSF) serine protease inhibitor. Cells were then lysed by sonication on ice for 7 min with a 2 s duty cycle at 80% amplitude (Qsonica Q125 with CL-18 probe). The lysate was centrifugated at 30,000*g* for 30 min at 4 °C and the supernatant was subjected to purification by two rounds of inverse transition cycling (ITC). In the first ITC round, (0.25–1 M) ammonium sulfate (Sigma-Aldrich) is used to suppress the transition temperature of PREs below room temperature. The sample is then centrifuged at 40 °C at 20,000*g* for 20 min. The pellet containing the PREs is resuspended in 10 mL of ice cold PBS^140^ pH 7.4 and then subjected to a cold round of centrifugation at 20,000*g* in a 4 °C centrifuge for 20 min, and the pellet was discarded. In the second ITC round, between 0.25 and 1.5 M NaCl (Sigma-Aldrich) was used to depress transition temperature and the same hot and cold centrifugation were performed. The protein pellet was resuspended in 5 mL ice-cold PBS^140^ pH 7.4 and extensively dialyzed against >10 L Milli-Q and finally lyophilized. For all PREs, two rounds of ITC were sufficient to remove >95% of impurities as determined by SDS-PAGE with yields between 5 and 40 mg L^–1^.

### Turbidity Measurements

Lyophilized PRE was dissolved to 25 μM by vortexing on ice in SBS^100^ (50 mM succinic acid + 100 mM NaCl + 1 mM DTT) for pH values < 6.0 and PBS^100^ (50 mM phosphate + 100 mM NaCl + 1 mM DTT) for pH > 6.0. These buffers were also chosen for their buffering capacity over a broad pH range (succinate p*K*_a_ = 4.25, phosphate p*K*_a_ = 7.21) and their relative insensitivity to temperature. Optical density measurements were performed on a UV–vis Evolution 220 (Thermo Fisher Scientific) using the Evolution Smart Thermostatted Linear 8-Cell Changer (Thermo Fisher Scientific). Temperature ramps were performed in micro volume plastic cuvettes while recording absorbance at 350 nm at a rate of 1 °C/min. For temperatures above 80 °C, 50 μL of quartz cuvettes were used. Absorbance data was normalized, and data was fitted using a sigmoid function in python using SciPy. The transition temperature (*T*_t_), is defined as the inflection point of the sigmoid fit to the turbidity data. The transition temperature-pH phase diagram was fitted using a Henderson–Hasselbach derived equation adopted from MacKay et al.^17^ Here, pH_t_ is the pH transition point, *T* is the temperature in °C, and *T*_c_ is a critical transition temperature in °C described by the extrapolated intersection of the concentration-dependent transition temperatures from ELPs of different lengths in pentamers, *L*. These curves intersect at a critical concentration, *C*_c_ in mM and interaction parameter *k* in °C pentamers, which is included to modulate the dependence of *T*_t_ on length and concentration. The subscripts pro and depro relate to the relative fractions of protonation of the ELP guest residues.
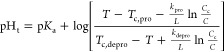
1

### Bioconjugation of AT590-PRE-*h*-46 and AT612Q-PRE-*h*-46

2.7 mg lyophilized PRE-*h*-46 was dissolved in 2.3 mL PBS^140^ pH 7.4 with a 10-fold molar excess TCEP (Sigma-Aldrich) and incubated for >15 min at room temperature to reduce disulfide bonds. Next, 1 mg of ATTO 590 maleimide (ATTO-TEC GmbH) or ATTO 612Q maleimide (ATTO-TEC GmbH) was dissolved in 200 μL DMSO (Sigma-Aldrich) and added dropwise to at approximately 10-fold molar excess. The reaction mixture was wrapped in aluminum foil and incubated in a rocker for 2 h at room temperature followed by overnight incubation at 4 °C. Following manufacturer’s instructions, a PD-10 column (GE Healthcare) was used to crudely separate excess dye from the conjugated protein. To quench remaining free dye, 1 mM DTT (Sigma-Aldrich) was added to the eluate, and the eluate was concentrated using 3.5 kDa spin filters (Amicon) at 4 °C to 1 mL. The sample was then further purified by size-exclusion chromatography on a Superdex 75 10/300 gl (GE Healthcare) equilibrated in PBS^140^ pH 7.4 on an Agilent 1260 Infinity II LC System (Agilent Technologies) at 0.6 mL/min flowrate (Figure S8). The fractions containing the conjugated protein were pooled, and *A*_280_ absorption was used to determine the final concentration of protein (MW = 26836.74 g/mol, ε_280_ = 5500 M^–1^ cm^–1^), taking into account the dye and quencher correction factors of 0.43 and 0.6 for AT590 and AT612Q, respectively. The degree of labeling was determined by UV–vis and found to be approximately 40, and 100% for AT590-PRE-*h*-46 and AT612Q-PRE-*h*-46 proteins, assuming molar extinction coefficients ε_AT590_ = 120,000 M^–1^ cm^–1^ and ε_612Q_ = 115,000 M^–1^ cm^–1^. The conjugated proteins were flash frozen in aliquots at −20 °C until use.

### Fluorescence “ON/OFF” Switch

AT590-PRE-*h*-46 and AT612Q-PRE-*h*-46 protein aliquots were thawed and mixed to prepare 25 μM. The samples were dialyzed overnight against 50 mL of SBS^100^ (50 mM succinate, 100 mM NaCl) of various pH (3.97, 4.18, 4.42, 4.57, 4.76, 5.02, 5.20, 5.42, 5.63, 5.87, 6.07) at 4 °C. First, samples were measured at 15 °C, below the *T*_t_, and then temperature was switched to 37 °C and measurements were performed after 5 min equilibration. Fluorescence emission spectra were obtained with a Cary Eclipse Fluorescence Spectrophotometer (Agilent Technologies) with excitation at 590 nm (5 nm excitation slit) and recorded emissions from 600 to 800 nm (2.5 nm emission slit) at medium scan rate.

### Bioconjugation of Cy5-PRE-*h*-36, Cy3-PRE-*h*-58, and FITC-PRE-*h*-41

Lyophilized PRE-*h*-36, PRE-*h*-58, and PRE-*h*-41 were dissolved at 1.5 mg/mL in PBS^140^ pH 7.0 in 500 μL. 10-fold molar excess freshly prepared TCEP (Sigma-Aldrich) was added and incubated for >15 min at room temperature. Next, 10-fold molar excess sulfo-Cy5 maleimide (Lumiprobe GmbH) sulfo-Cy3 maleimide (Lumiprobe GmbH) and FITC maleimide (Lumiprobe GmbH) from a 10 mg/mL stock in DMSO (Sigma-Aldrich) was added dropwise to the proteins to yield Cy5-PRE-*h*-36, Cy3-PRE-*h*-58, and FITC-PRE-*h*-41. The reaction mixture was wrapped in aluminum foil and incubated in a rocker for 2 h at room temperature followed by overnight incubation at 4 °C. Excess dye was quenched by addition of 1 mM DTT and extensively removed by multiple rounds of dilution of the labeled conjugate with PBS^140^ followed by 10 kDa spin concentrations (Amicon), until no absorbance could be measured by UV–vis in the eluate.

### pH Calibration of GDL Hydrolysis

Gradual acidification over time can be achieved by the hydrolyzation of GDL (Sigma-Aldrich) into gluconic acid. To establish a pH versus time calibration curve, 300 mg GDL was dissolved in 1 mL and immediately added to a stirred beaker of 19 mL PBS^100^ pH 7.4 at a final concentration of 15 mg/mL (84.2 mM) with a calibrated pH probe inserted, and pH was recorded over time with intervals of 5 s for 4 h at room temperature (21 °C). The experiment was repeated 3 times.

### Fluorescence Microscopy of pH-Triggered Condensate Formation

A solution of 20 μL, with a final concentration of 25 μM PRE-*h*-46 was used for microscopic visualization. For fluorescent imaging, the PRE-*h*-46 was doped with 5% fluorescently labeled AT590-PRE-*h*-46 (23.75 μM unlabeled and 1.25 μM labeled). Multichannel imaging of the ELP mixture was made feasible by fluorescently labeling ELPs with fluorophores of different emission spectra. Two pairs were chosen to demonstrate this and: (i) Cy5-PRE-*h*-36 and Cy3-PRE-*h*-58 (ii) Cy5-PRE-*h*-36 and FITC-PRE-*h*-41. The final concentration of each ELP in the mixture was 25 μM with 10% fluorescently labeled ELP (22.5 μM unlabeled and 2.5 μM labeled). To trigger condensation, 1 μL freshly dissolved GDL 300 mg/mL was added (15 mg/mL final concentration) to 19 μL of PRE mixture in a custom polydimethylsiloxane (PDMS) well prepared as previously described.^34^ Time-lapse images were acquired using a Prime BSI Express sCMOS camera connected to a Nikon-Ti2-Eclipse inverted fluorescence microscope, equipped with a pE-300 ultra illumination light source. The dynamics of PRE condensate formation were acquired by using a Nikon Plan Apo 100×/1.45 NA oil objective. Images for FITC-PRE-*h*-41 was detected using a 482/35 nm excitation filter and a 536/40 nm emission filter (Semrock), Cy3-PRE-*h*-58 was detected using a 543/22 nm excitation filter and a 593/40 nm emission filter (Semrock). Cy5-PRE-*h*-36 was detected by using a 628/40–25 nm excitation filter and a 692/40–25 nm emission filter (Semrock). The PRE samples were illuminated at 2–5% laser intensity, exposure time was adjusted between 5 and 20 ms, and time-lapse images were taken each 15 s. Acquired images were analyzed using Fiji ImageJ 1.52 software.

### Synthetic Cell Production with Intracellular Fluorescent PREs Using Microfluidics

The water-in-oil-in-water double emulsion droplets were generated using two cross-flow focusing junctions, using a PDMS-based microfluidic device (Figure S6). The first junction generated water-in-oil single emulsions where the inner aqueous phase consisted of PRE-*h*-36 and Cy3-PRE-*h*-58 dissolved in PBS^100^ and the oil phase was composed of fluorinated oil HFE-7500 with 2% Pico surf (Sphere fluidics). At the second junction, double emulsions were formed and stabilized with PBS^100^ and 1% Tween-20 (Sigma) surfactant. The droplets generated were collected and stored in a glass vial at 4 °C. During experimentation, 5 μL of double emulsion dispersion and 5 μL of PBS^100^ at pH 7.4 was added to a custom PDMS well.^34^ To trigger PRE condensation, 10 μL of PBS^100^ at pH 2 was added to the solution and images were taken at an interval of 15 s.

## Supporting Information Available

Additional details on protein purification, fluorescence spectroscopy, turbidity measurements, microfluidic production, and sequences of proteins designed in this study (PDF)
