# Supplementary material for: pH-Responsive Elastin-Like Polypeptide Designer Condensates
Source: ACS Appl Mater Interfaces. 2023 Sep 14;15(38):45336–44. doi: 10.1021/acsami.3c11314 (PMC10540133; doi:10.1021/acsami.3c11314)
Supplement: Supplementary file 1 — am3c11314_si_001.pdf [file am3c11314_si_001.pdf]

Supporting Information

pH-responsive elastin-like polypeptide designer condensates

Robbert J. de Haas, Ketan A. Ganar, Siddharth Deshpande and Renko de Vries*

Department of Physical Chemistry and Soft Matter, Wageningen University and Research, 6708 WE, Wageningen, The Netherlands.

* Corresponding author. Email: renko.devries@wur.nl

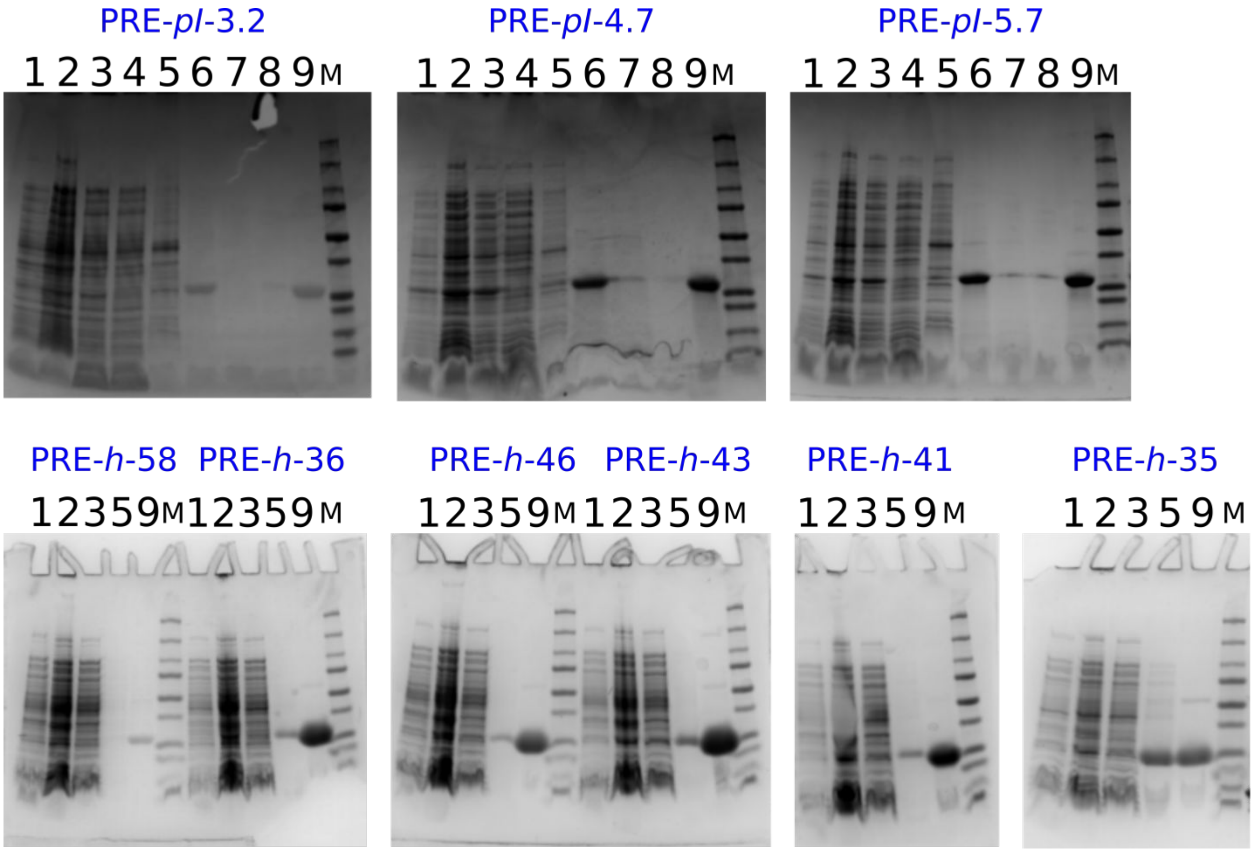

Figure S1 - Sodium dodecyl-sulfate polyacrylamide gel electrophoresis (SDS-PAGE) gels of recombinant protein purification in *E.coli*. Proteins are purified using two successive rounds of Inverse Temperature Cycling (ITC) method. Lanes: (1) induced cells (2) cell lysate (3) cell supernatant (4) ITC round 1 hot supernatant (5) ITC round 1 hot pellet (6) ITC round 1 cold supernatant (7) ITC round 2 hot supernatant (8) ITC round 2 cold pellet (9) ITC round 2 cold supernatant. Final purified product is present in lane (9). (M) is a Precision Plus Protein Dual Color Standard (bands top to bottom: 250, 150, 100, 75, 50, 37, 25, 20, 15, 10 kDa). Note for PRE-h-35, PRE-h41, PRE-h-43, PRE-h-46, PRE-h-36 and PRE-h-58 sample (8) was not analyzed by SDS-PAGE.

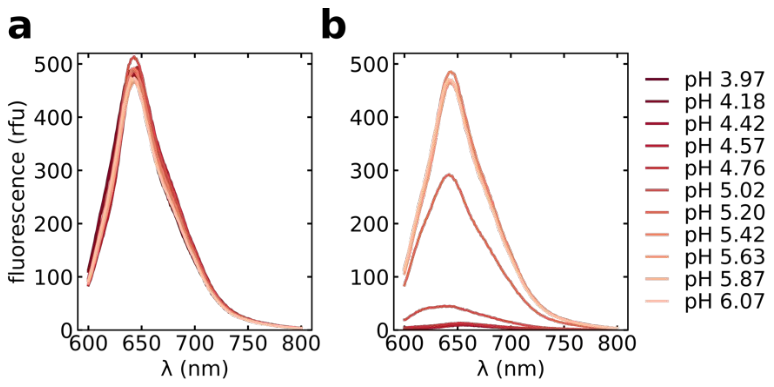

Figure S2 - Fluorescence spectra of AT590-PRE-*h*-46 and AT612Q-PRE-*h*-46 at 25 μM dialyzed in SBS^{100} (50 mM succinate + 100 mM NaCl) at pre-set pH. **(a)** fluorescence spectra at 15 °C. **(b)** fluorescence spectra at 37°C after 5 minute incubation. At 37°C a >50-fold intensity reduction is observed over a ~0.3 pH difference due to coacervation leading to AT590 fluorescence quenching.

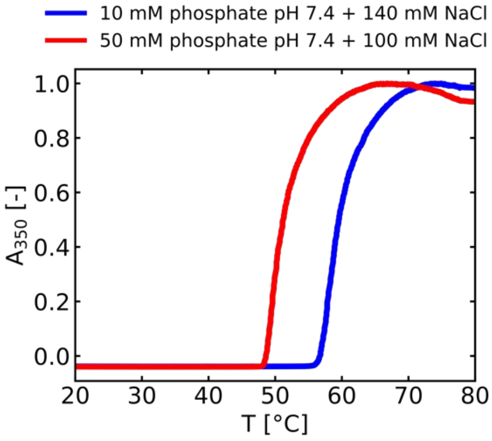

Figure S3 - Turbidity measurements of PRE-*h*-43 at 25 µM in phosphate-saline buffers of approximately equal ionic strength, but varying in phosphate and NaCl concentration. A transition temperature (T_t) shift of approxiately 10 °C is observed, can be partly attributed to a higher Na^+ ion concentration.

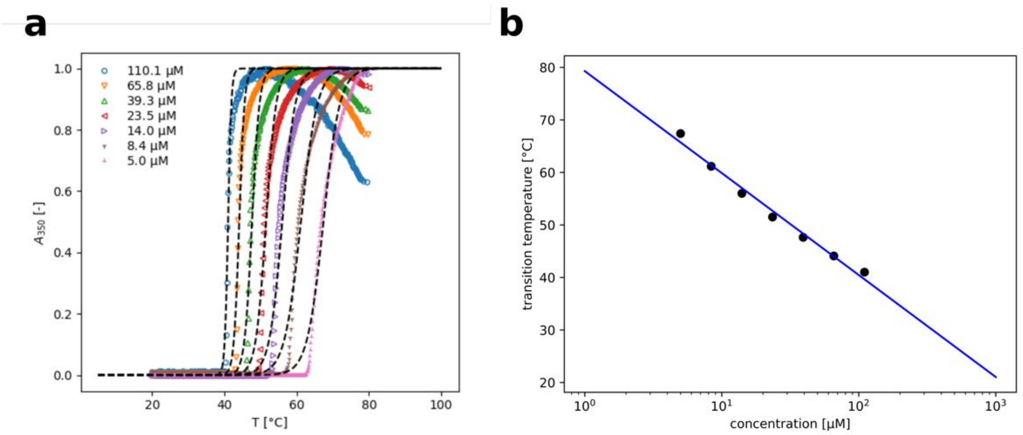

Figure S4 - (a) Turbidity measurements of PRE-*h*-43 at various protein concentrations.Black dotted line are sigmoidal fits through the data. (**b)** Transition temperature (T_t) – defined as midpoints of the sigmoid plots – are plotted over the PRE-*h*-43 polymer concentration.

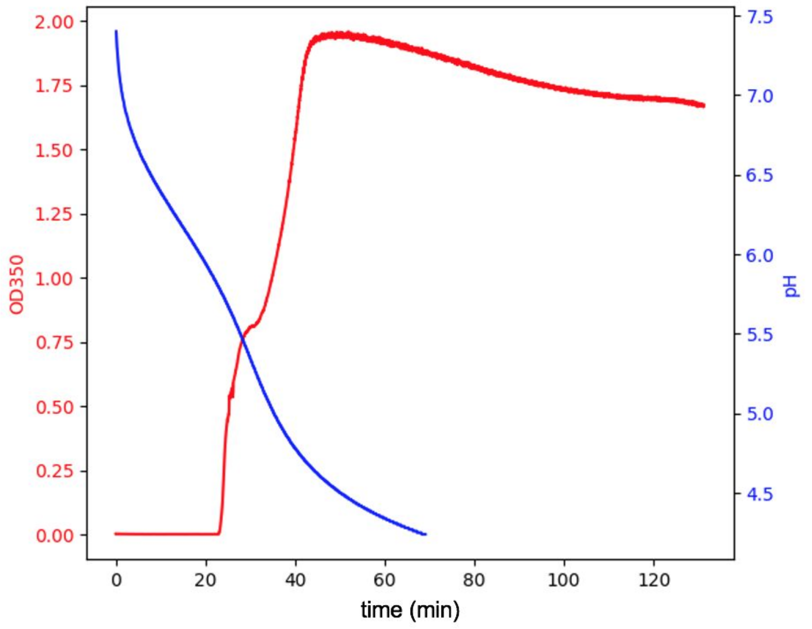

Figure S5 - Turbidity measurement of 25 µM PRE-*h*-43 in presence of 14.3 mg/mL GDL at constant temperature of 21 °C. In blue y-axis the GDL calibration is plotted and the OD_{350} is recorded in the red y-axis as a measure for turbidity (indicating condensation). From the data it can be observed that complete condensation can take >20 min indicating that the dynamics of pH-triggered condensation are relatively slow compared to temperature-driven condensation (where condensation typically completes within ~ 3 min with a 1 °C /min temperature ramp).

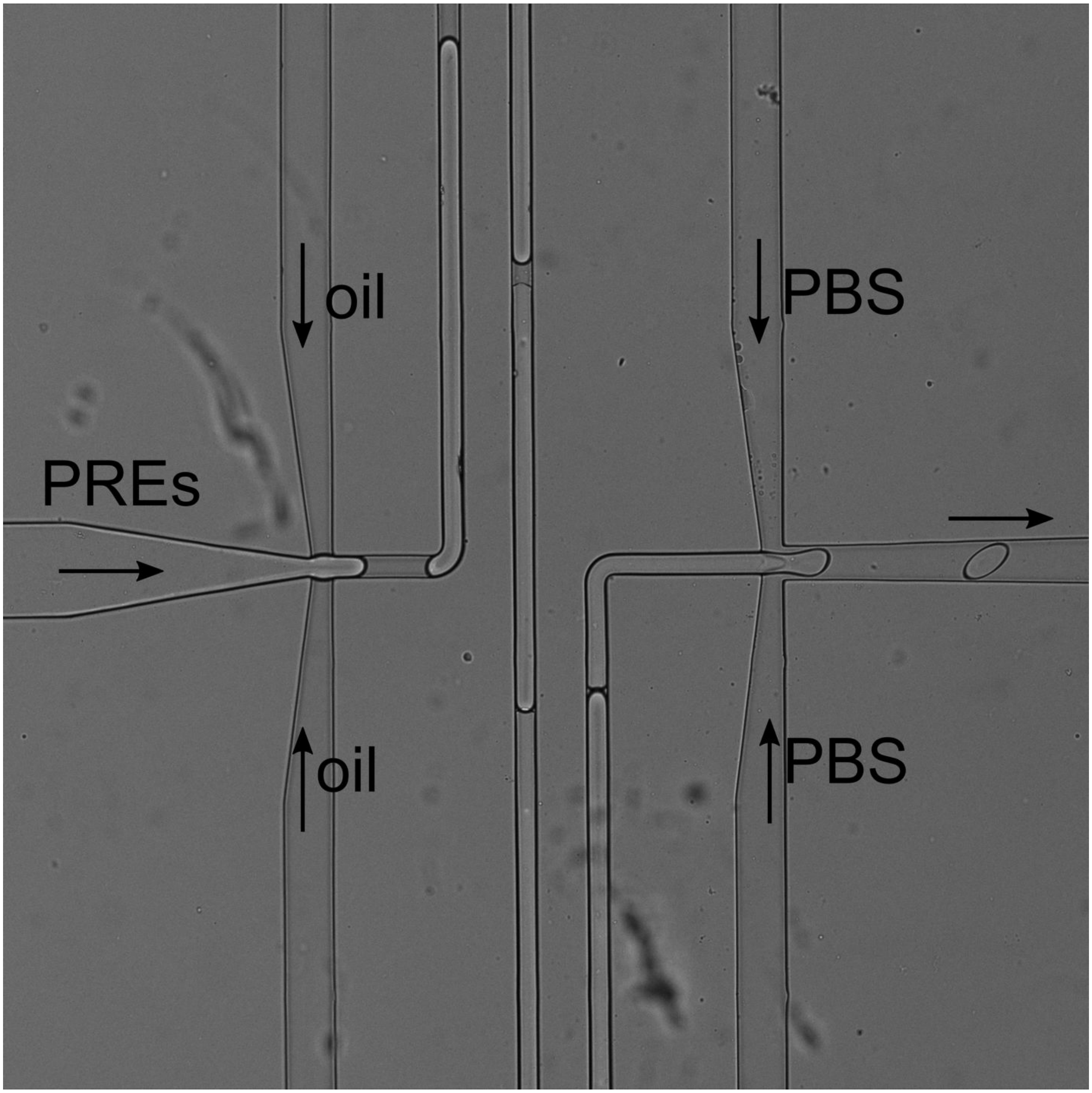

Figure S6 - Brightfield image of double emulsion (water-oil-water) droplet production using PDMS-based microfluidic setup. The inner aqueous stream composed of PREs flows through the flow-focusing junction, forming water-in-oil single emulsions. The single emulsions flow through the serpentine loop and get pinched-off at the second junction by a second cross-flow of surfactant-containing PBS solution, leading to water-in-oil-in-water double emulsion droplets.

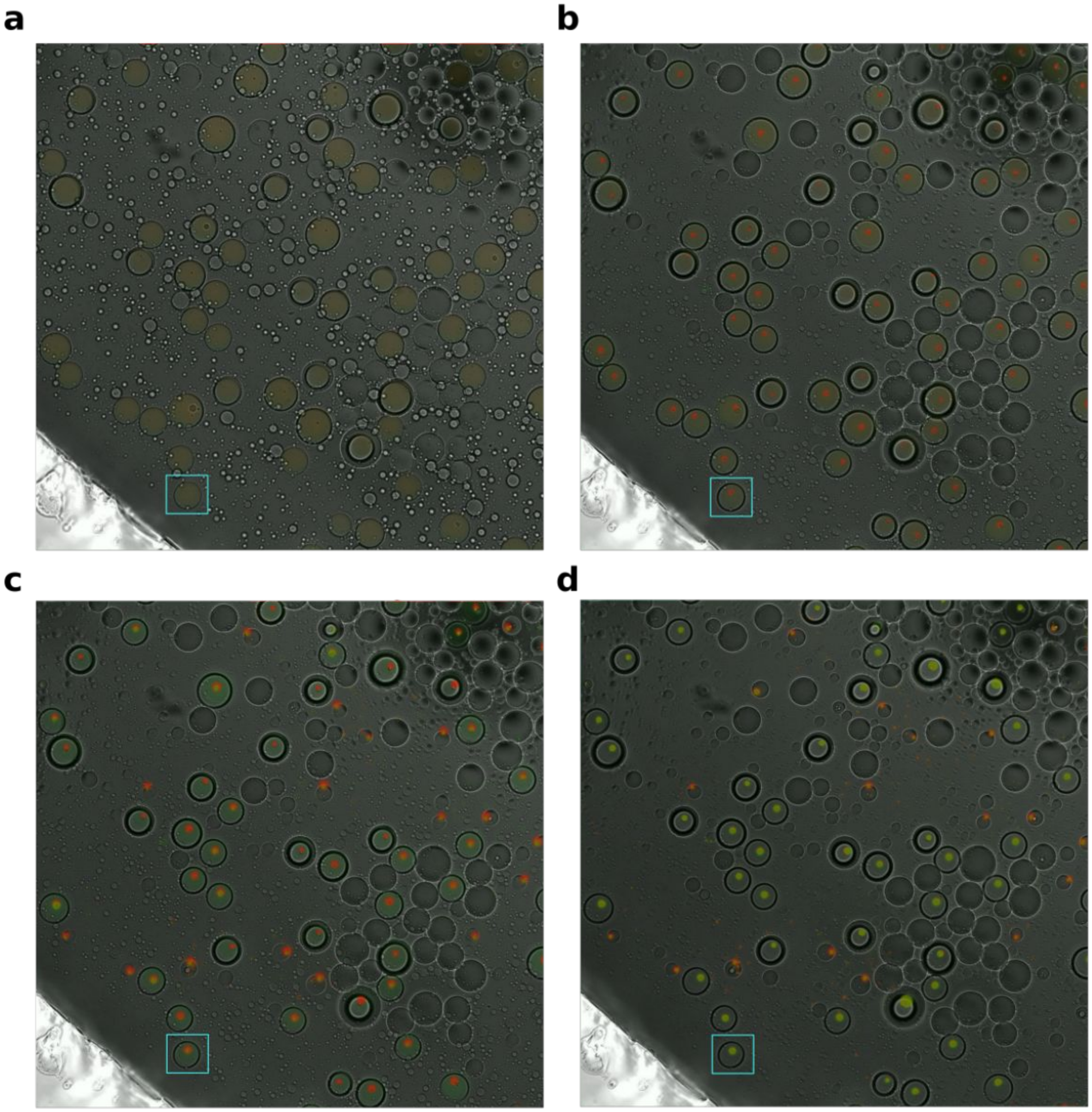

Figure S7 - Full views of sequential (mixed) condensate formation in microcompartments water-oil-water droplets. Cyan colored highlights the square showing **Figure 7b** of the main text. **(a)** t_0, **(b)** t_1 **(c)** t_2, **(d)** t_{end}. The droplets observed in the background are unwanted oil droplets formed as a by-product of double emulsion production.

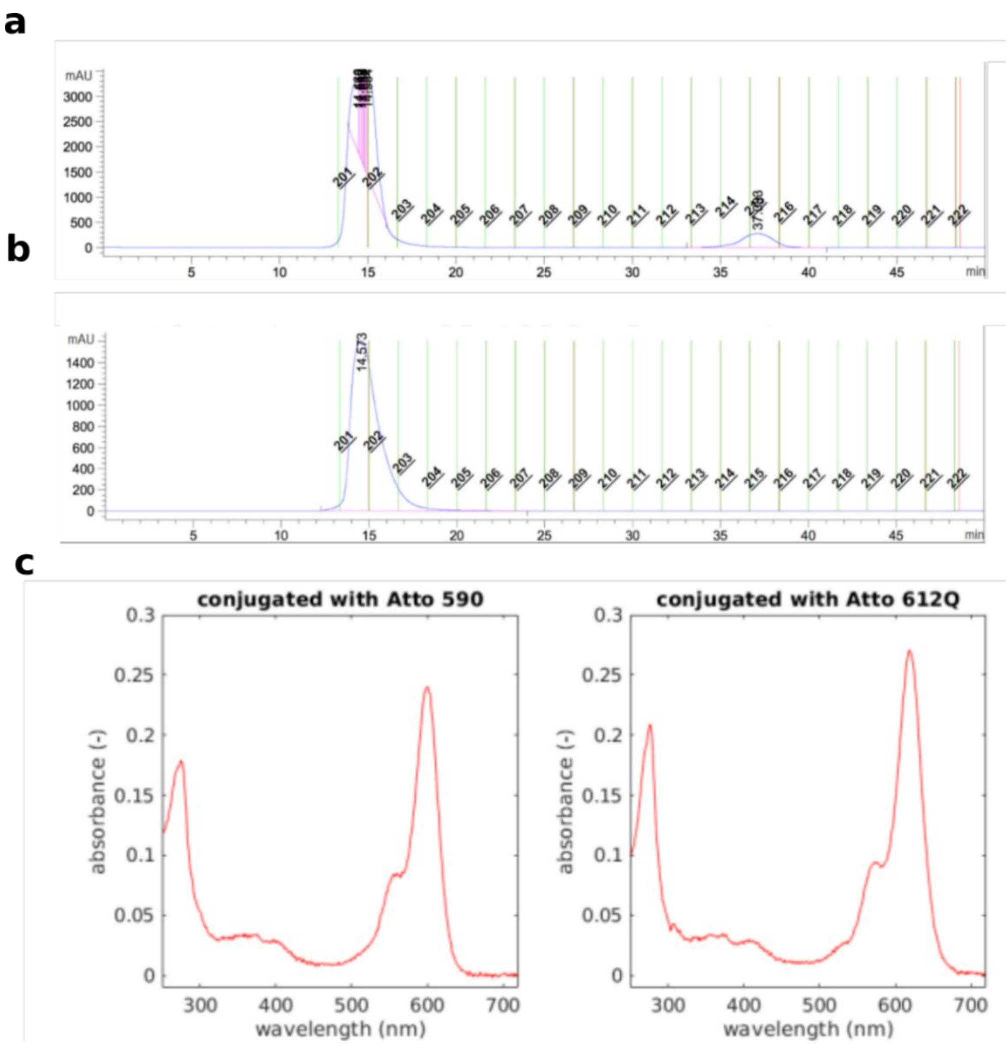

Figure S8 - (a) Preparative HPLC trace of AT590-PRE-*h*-46 on a Superdex 75 10/300 (GE Healthcare) column at 0.6 mL/min flowrate. The minor peak at ~37 minutes corresponds to excess free-dye. (**b)** Preparative HPLC trace of AT612Q-PRE-*h*-46 on a Superdex 75 10/300 (GE Healthcare) column at 0.6 mL/min flowrate. Y-axis shows absorbance at 590 nm in mAU, x-axis shows time in minutes. **(c)** UV-VIS spectra of post-HPLC purified AT590-PRE-*h*-46 and AT612Q-PRE-*h*-46.

Table S1. PRE protein sequences. Trailer sequence WPC is added (tryptophan (W) for protein A_{280} adsorption quantification, proline (P) as a spacer, and cysteine (C) for fluorescent dye conjugation using thiol-maleimide chemistry).

**Design name**	**Protein sequence**
PRE-*pI*-3.2	MGVGVPGIGVPGIGVPGEGVPGIGVPGVGVPGIGVPGIGVPGEGVPGIGV<br>PGVGVPGIGVPGIGVPGEGVPGIGVPGVGVPGIGVPGIGVPGEGVPGIGV<br>PGVGVPGIGVPGIGVPGEGVPGIGVPGVGVPGIGVPGIGVPGEGVPGIGV<br>PGVGVPGIGVPGIGVPGEGVPGIGVPGVGVPGIGVPGIGVPGEGVPGIGV<br>PGVGVPGIGVPGIGVPGEGVPGIGVPGVGVPGIGVPGIGVPGEGVPGIGV<br>PGVGVPGIGVPGIGVPGEGVPGIGVPGVGVPGIGVPGIGVPGEGVPGIGV<br>PG
PRE-*pI*-4.7	MGIGVPGIGVPGVGVPGHGVPGIGVPGIGVPGVGVPGEGVPGIGVPGIGV<br>PGVGVPGHGVPGIGVPGIGVPGVGVPGEGVPGIGVPGIGVPGVGVPGEG<br>VPGIGVPGIGVPGVGVPGHGVPGIGVPGIGVPGVGVPGEGVPGIGVPGIG<br>VPGVGVPGHGVPGIGVPGIGVPGVGVPGEGVPGIGVPGIGVPGVGVPGE<br>GVPGIGVPGIGVPGVGVPGHGVPGIGVPGIGVPGVGVPGEGVPGIGVPGI<br>GVPGVGVPGHGVPGIGVPGIGVPGVGVPGEGVPGIGVPGIGVPGVGVPG<br>EGVPG
PRE-*pI*-5.7	MGIGVPGIGVPGVGVPGHGVPGIGVPGIGVPGVGVPGEGVPGIGVPGIGV<br>PGVGVPGHGVPGIGVPGIGVPGVGVPGHGVPGIGVPGIGVPGVGVPGEG<br>VPGIGVPGIGVPGVGVPGHGVPGIGVPGIGVPGVGVPGEGVPGIGVPGIG<br>VPGVGVPGHGVPGIGVPGIGVPGVGVPGHGVPGIGVPGIGVPGVGVPGE<br>GVPGIGVPGIGVPGVGVPGHGVPGIGVPGIGVPGVGVPGEGVPGIGVPGI<br>GVPGVGVPGHGVPGIGVPGIGVPGVGVPGHGVPGIGVPGIGVPGVGVPG<br>EGVPG
PRE-*h*-58	MGIGVPGIGVPGIGVPGEGVPGIGVPGIGVPGIGVPGEGVPGIGVPGVGVP<br>GIGVPGHGVPGIGVPGVGVPGIGVPGEGVPGIGVPGIGVPGIGVPGEGVP<br>GIGVPGIGVPGIGVPGEGVPGIGVPGIGVPGIGVPGEGVPGIGVPGVGVPG<br>IGVPGHGVPGIGVPGVGVPGIGVPGEGVPGIGVPGIGVPGIGVPGEGVPGI<br>GVPGIGVPGIGVPGEGVPGIGVPGIGVPGIGVPGEGVPGIGVPGVGVPGIG<br>VPGHGVPGIGVPGVGVPGIGVPGEGVPGIGVPGIGVPGIGVPGEGVPGW<br>PC
PRE-*h*-36	MGVGVPGYGVPGEGVPGVGVPGYGVPGVGVPGEGVPGYGVPGVGVPG<br>YGVPGHGVPGYGVPGVGVPGEGVPGYGVPGVGVPGYGVPGEGVPGVG<br>VPGYGVPGVGVPGYGVPGEGVPGVGVPGYGVPGVGVPGEGVPGYGVP<br>GVGVPGYGVPGHGVPGYGVPGVGVPGEGVPGYGVPGVGVPGYGVPGE<br>GVPGVGVPGYGVPGVGVPGYGVPGEGVPGVGVPGYGVPGVGVPGEGV<br>PGYGVPGVGVPGYGVPGHGVPGYGVPGVGVPGEGVPGYGVPGVGVPG<br>YGVPGEGVPGVGVPGYGVPGWPC

PRE-*h*-46	MGVGVPGFGVPGEGVPGVGVPGFGVPGVGVPGEGVPGFGVPGVGVPG<br>FGVPGHGVPGFGVPGVGVPGEGVPGFGVPGVGVPGFGVPGEGVPGVG<br>VPGYGVPGVGVPGFGVPGEGVPGVGVPGFGVPGVGVPGEGVPGFGVP<br>GVGVPGFGVPGHGVPGFGVPGVGVPGEGVPGFGVPGVGVPGFGVPGE<br>GVPGVGVPGYGVPGVGVPGFGVPGEGVPGVGVPGFGVPGVGVPGEGV<br>PGFGVPGVGVPGFGVPGHGVPGFGVPGVGVPGEGVPGFGVPGVGVPG<br>FGVPGEGVPGVGVPGFGVPGWPC
PRE-*h*-43	MGFGVPGEGVPGVGVPGFGVPGFGVPGVGVPGEGVPGFGVPGFGVPG<br>VGVPGHGVPGFGVPGFGVPGVGVPGEGVPGFGVPGFGVPGVGVPGEG<br>VPGFGVPGFGVPGEGVPGVGVPGFGVPGFGVPGVGVPGEGVPGFGVP<br>GFGVPGVGVPGHGVPGFGVPGFGVPGVGVPGEGVPGFGVPGFGVPGV<br>GVPGEGVPGFGVPGFGVPGEGVPGVGVPGFGVPGFGVPGVGVPGEGV<br>PGFGVPGFGVPGVGVPGHGVPGFGVPGFGVPGVGVPGEGVPGFGVPG<br>FGVPGVGVPGEGVPGFGVPGWPC
PRE-*h*-35	MGFGVPGEGVPGFGVPGFGVPGFGVPGVGVPGEGVPGFGVPGFGVPG<br>FGVPGHGVPGFGVPGFGVPGFGVPGEGVPGFGVPGFGVPGVGVPGEG<br>VPGFGVPGFGVPGEGVPGFGVPGFGVPGFGVPGVGVPGEGVPGFGVPG<br>FGVPGFGVPGHGVPGFGVPGFGVPGFGVPGEGVPGFGVPGFGVPGVGV<br>PGEGVPGFGVPGFGVPGEGVPGFGVPGFGVPGFGVPGVGVPGEGVPGF<br>GVPGFGVPGFGVPGHGVPGFGVPGFGVPGFGVPGEGVPGFGVPGFGVP<br>GVGVPGEGVPGFGVPGWPC
PRE-*h*-41	MGFGVPGEGVPGVGVPGFGVPGFGVPGVGVPGEGVPGFGVPGFGVPG<br>VGVPGHGVPGFGVPGFGVPGVGVPGEGVPGFGVPGFGVPGVGVPGEG<br>VPGFGVPGFGVPGEGVPGVGVPGFGVPGFGVPGVGVPGEGVPGFGVP<br>GFGVPGVGVPGHGVPGFGVPGFGVPGVGVPGEGVPGFGVPGFGVPGV<br>GVPGEGVPGFGVPGFGVPGEGVPGVGVPGFGVPGFGVPGVGVPGEGV<br>PGFGVPGFGVPGVGVPGHGVPGFGVPGFGVPGVGVPGEGVPGFGVPG<br>FGVPGVGVPGEGVPGFGVPGWPC
